# Converging Modalities Ground Abstract Categories: The Case of Politics

**DOI:** 10.1371/journal.pone.0060971

**Published:** 2013-04-10

**Authors:** Ana Rita Farias, Margarida V. Garrido, Gün R. Semin

**Affiliations:** 1 Instituto Universitário de Lisboa (ISCTE-IUL), Cis-IUL, Lisboa, Portugal; 2 Faculty of Social and Behavioural Sciences, Utrecht University, Utrecht, The Netherlands; 3 Faculty of Social and Behavioural Sciences, Utrecht University, Utrecht, The Netherlands; 4 College of Social Sciences and Humanities, Koç University, Istanbul, Turkey; Colorado State Univeresity, United States of America

## Abstract

Three studies are reported examining the grounding of abstract concepts across two modalities (visual and auditory) and their symbolic representation. A comparison of the outcomes across these studies reveals that the symbolic representation of political concepts and their visual and auditory modalities is convergent. In other words, the spatial relationships between specific instances of the political categories are highly overlapping across the symbolic, visual and auditory modalities. These findings suggest that abstract categories display redundancy across modal and amodal representations, and are multimodal.

## Introduction

The current research trend on embodiment demonstrates the diverse ways in which our representations of concepts result from embodied experiences that are activated during a concept’s processing (e.g., [1]–[4]). This trend has emerged in contrast to views arguing that the meaning of symbols is non-perceptual and derived by their relation to other amodal symbols (e.g., [5]–[8]).

Recent studies deriving from the embodiment perspective show that language comprehension involves the simulation and recruitment of neural systems used for perception, action, and emotion (e.g., [9]–[13]). Considerable evidence supporting the embodied grounding of concrete concepts indicates that conceptual processing is facilitated by congruencies between movements implied by the concept and response movements, with congruent and incongruent spatial arrangements influencing response times or gaze movements (e.g., [14]–[20]).

This debate, has taken place predominantly with reference to concrete concepts, and has not touched another burgeoning area namely the grounding of abstract concepts, (cf. [21] for a review). Abstract social categories such as power ([22]), or categories related to affect (e.g., [23]) or time (e.g., [24]) were shown to rely on spatial representations that provide relational structure to these domains (cf. [25]–[26]). These studies not only show evidence for embodiment in conceptual processing, but may even give the impression that there is not much more to conceptual processing than the activation of embodied representations.

A more recent and conciliatory approach has started to acknowledge that conceptual processing is both linguistic and embodied (e.g., [27]–[32]). For instance, there is evidence that language encodes embodied relations ([33]–[34]). Consequently, language users might rely on language, on embodied relations, or on both and concepts can be represented in more than one modality.

While these demonstrations have been extremely valuable in opening new ways of thinking about how we represent different categories they have not informed us about whether the relational structure of a category holds across modalities. Nor has this work established the interface between symbolic representations and modal ones. Consequently, the question about the relationship between the relational structure of symbolic and visual as well as auditory representations of an abstract category has not been systematically examined.

The three studies reported in this paper were based on the argument that the relational structure representing an abstract category in one modality (e.g., visual) should overlap with the relational structure in a second modality (e.g., auditory). Moreover, the structure obtained in the two modalities should not diverge from the relational structure that holds in the symbolic representation of the category. We shall present the implications of this research for the ongoing discussion on the embodiment of concrete and abstract concepts (e.g., [35]–[36], [28]–[29], [37], [21]) in the concluding section of this paper.

## Overview

In the research we report, we use the political categories of left and right, demonstrably represented horizontally in space [38], [39]. Study 1 examined the semantic properties of politically-charged words, namely the degree to which they represent a socialist or a conservative ideology. This gave us a graded anchoring of each term on a conservatism-socialism semantic dimension. Study 2 examined how these politically-charged words are visually distributed in space by analyzing how participants distribute them on a horizontal line. This furnished a graded visual spatial ordering of the same words. In Study 3 these words were presented over headphones with participants deciding on which channel the word was louder. This provided an auditory spatial representation of the same politically-charged words. We then examined the degree of overlap between the audio and visual anchoring of the concepts and their semantic counterpart.

The general hypothesis was that spatially grounded political terms should have a very significant degree of overlap across semantic, visual and auditory representations. Support for this hypothesis would suggest that abstract categories such as politics are multimodally grounded.

## Study 1: The Semantics of Politics

The purpose of this study was to obtain a graded semantic anchoring of each term of the socialism-conservatism dimension, thereby revealing the semantic spatial distances between the terms.

### Method

#### Participants

Fifty-four university students (42 females, *M_age_* = 24.22, *SD = *6.70) voluntarily participated in this study. All procedures were executed in compliance with relevant laws and institutional guidelines and were approved by the ethics committee of the Instituto Universitário de Lisboa (ISCTE-IUL). All participants gave written informed consent for their participation. All participants gave written informed consent for their participation.

#### Stimulus Materials and procedure

A first group of participants (N = 65) was asked to generate 20 words associated with the concepts of socialism (10) and conservatism (10). The final list of 123 political-related words was rated by an independent group of participants (N = 54) regarding their political meaning on a seven-point scale (socialism to conservatism). Additionally, participants evaluated the valence of the words to dismiss confounds between valence and the horizontal dimension (see [40]). Finally, they provided information regarding their general political awareness (interest, engagement, knowledge) and their own political orientation.

### Results

Socialism and conservatism-referent words were selected, based on: (1) the confidence intervals of each word (upper bounds below 3 = “socialist”; lower bounds above 5 = “conservative”); (2) the socialism and conservatism-referent words were overall neutral in valence; (3) their ratings on political meaning and valence were independent of participants’ political awareness and political orientation.

The socialism-related words were: *Communism* (*M = *1.90, *SD = *1.43), *Revolution* (*M = *2.50, *SD = *1.38), *Union* (*M = *2.67, *SD = *1.40), *Proletariat* (*M = *2.69, *SD = *1.29), and *Demonstration* (*M = *2.73, *SD = *1.12); the conservatism-related words were: *Stockmarket* (*M = *4.88, *SD = *1.47), *Consumerism* (*M = *4.92, *SD = *1.44), *Profit* (*M = *4.92, *SD = *1.22), *Wealth* (*M = *4.96, *SD = *1.27), and *Colonialism* (*M = *5.08, *SD = *1.64). The difference between the political meaning scores’ of socialism (*M = *2.52, *SD = *.94) and conservatism-referent words (*M = *4.95, *SD = *.99) was highly significant, *t*(52) = 10.05, *p*<.001.

## Study 2: Spacing Out Politics

In a visual positioning task participants were asked to place socialism and conservatism-referent words on a horizontal line presented on the computer-monitor. We predicted that conservatism-referent words would be placed more to the right and socialism-referent words more to the left.

### Method

#### Participants

Seventy-nine university students (50 females; *M_age_* = 23.42, *SD = *6.06) participated in this study for partial course credit. All procedures were executed in compliance with relevant laws and institutional guidelines and were approved by the ethics committee of the Instituto Universitário de Lisboa (ISCTE-IUL). All participants gave written informed consent for their participation.

#### Stimulus materials

The 10 political words obtained in the first study were used along with three neutral words introduced to reduce the political salience of the stimuli and to establish a baseline of comparison with the political words.

#### Procedure

Participants were seated in front of a computer monitor and asked to place the stimulus words on a horizontal line. The ends of this line were unmarked and there was only an indicator of the line midpoint. The words were presented in a random order on the center of the monitor. Participants’ clicked on the line with the mouse to mark the position that they thought best suited each word. After the placement task the word disappeared and a second word was presented, and so on.

### Results and Discussion

The horizontal line was transformed to represent a scale ranging from 0 (socialist) to 100 (conservative). The average spatial position scores for the three word sets differed significantly, *F*(2,156) = 11.72, *p<*.001, η_p_^2^ = .131, indicating, as predicted, that conservatism-referent words were placed more to the right (*M = *54.04, *SD = *16.64) of the horizontal line compared to socialism-referent words (*M = *39.95, *SD = *19.22) with the neutral words in between (*M = *47.65, *SD = *13.22). The comparisons between socialism vs. neutral, *t*(78) = −2.68, *p*<.009; conservative vs. neutral, *t*(78) = 3.16, *p*<.002; socialism vs. conservative *t*(78) = −3.89, *p*<.001, were all significant. Further analysis indicated that socialism and conservatism-referent words differed significantly from the scale midpoint, *t*(78) = −4.65, *p<*.001, and *t*(78) = 2.16, *p<*.034, respectively. The absolute distance of socialism-related words was skewed more to the left of the midpoint (*M = *10.05, *SD = *19.22) than conservatism-related words were skewed to the right (*M = *4.04, *SD = *16.64). This difference was significant, *t*(78) = 3.89, *p*<.001, and may correspond to a bias derived from the habitual writing direction, namely from left to right [41]–[42].

**Figure 1:**
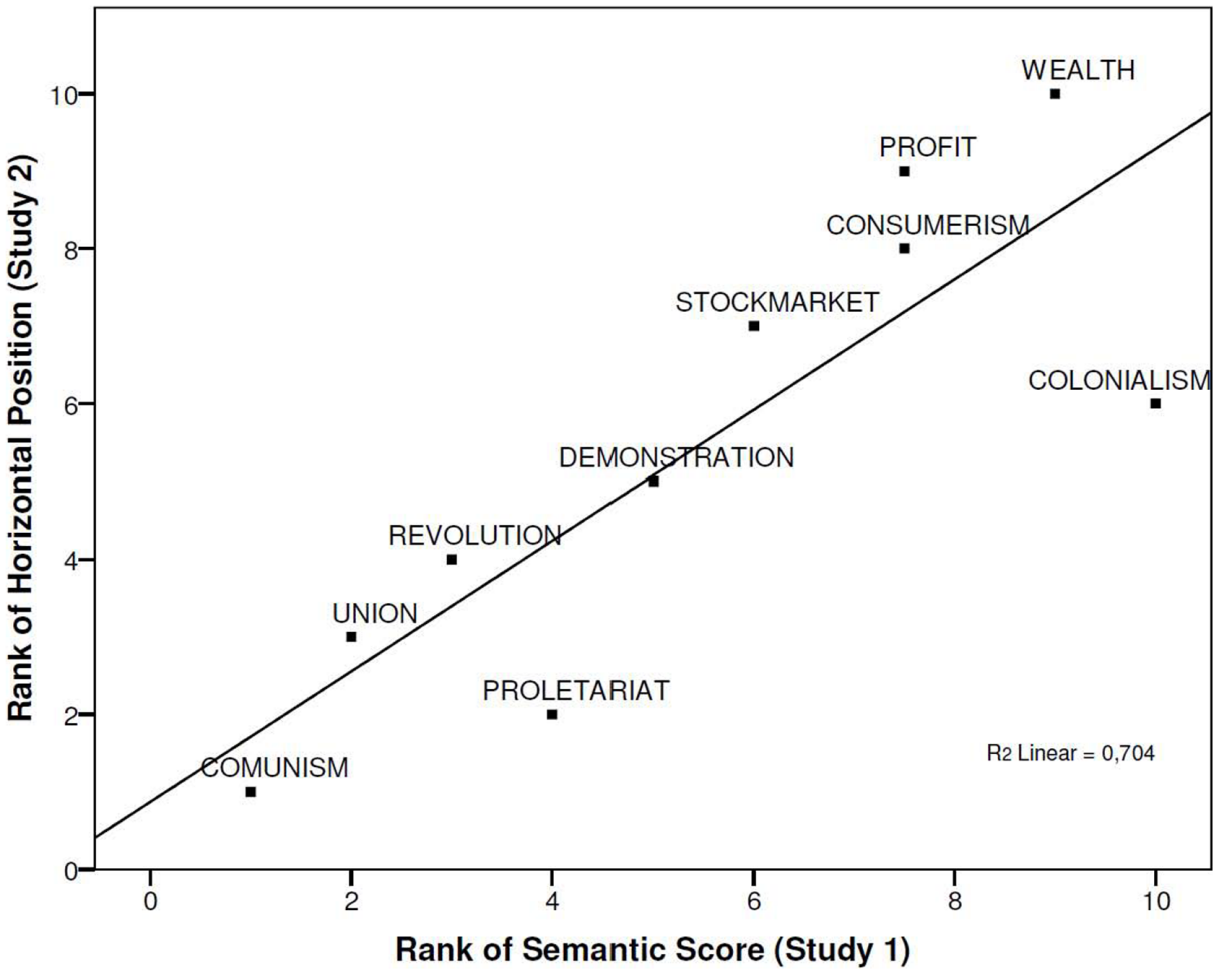
Ranked semantic judgments of the political stimuli in Study 1 plotted against their ranked horizontal position in Study 2.

Finally, we tested the convergence between the semantic and visual representations with a linear regression on the spatial ranked scores of the political-referent words in this study with the ranked semantic ratings obtained in Study 1 as predictor. As expected, the systematic order of the political meaning of the stimuli predicted their horizontal spatial position observed in the visual task, β = .839, *t*(9) = 4.36, *p*<.002 (R^2^ = .704). Thus, the more conservative the political meaning of the words is, the more the words were placed to the right and the more socialist the meaning the higher the bias in placing them towards the left (Figure 1). These results indicate that the semantic and visual grounding of words associated with distinct political positions display a remarkable overlap. Does this pattern of results generalize to another modality? In order to answer this question we turned to examining the same spatial-semantic ordering in the auditory modality.

## Study 3: The Sound of Politics

Study 3 employed an auditory disambiguation task (cf. [43]). Participants were presented with a list of words over headphones and had to indicate on which ear, each presented word sounded louder. Consistent with the earlier two studies, we predicted that, on critical trials, namely when a word was presented equally loud on both auditory channels, conservatism-referent words would be disambiguated more to the right than socialism-referent words. The neutral words were expected to be equally disambiguated to the left or right ear.

### Method

#### Participants

One hundred fourteen university students (55 females; *M_age_* = 20.41, *SD = *2.19) participated in this study for partial course credit. All procedures were executed in compliance with relevant laws and institutional guidelines and were approved by the ethics committee of the Instituto Universitário de Lisboa (ISCTE-IUL). All participants gave written informed consent for their participation.

#### Stimulus materials and procedure

The words used in Study 1 were converted to sound files using a text-to-speech application (AcapelaBox).

Participants seating in front of a computer-monitor, and wearing headphones were asked to indicate on which ear each word presented was louder by pressing a response key. The keys were aligned vertically and were counterbalanced. In total there were 78 trials. In critical trials (39) each word was presented three times equally loud on both auditory channels. In the remaining trials words were presented randomly with different volumes (100%, 50%) to the left and the right auditory channels.

### Results and Discussion

Because left and right auditory judgments are mutually dependent, we calculated the average percentage of times each critical word was judged to be louder in the right ear, with.50 indicating an equal number of left and right channel judgments, and 1.00 indicating only right-ear judgments. Response key assignment did not influence the results.

As predicted, a within-participants ANOVA with three levels (conservatism vs. neutral vs. socialism) revealed that, on critical trials, average right ear disambiguation differed as a function of the words’ political meaning; *F*(2,226) = 10.19 *p<*.001, η_p_^2^ = .083.

The expected linear trend indicated that participants were more likely to judge conservatism-referent words to be louder on the right ear (*M = *.60; *SD = *.23), than neutral (*M = *.56; *SD = *.26), than socialism-referent words (*M = *.52; *SD = *.22). The comparisons between socialism vs. neutral, *t*(113) = −2.37, *p*<.020; conservatism vs. neutral, *t*(113) = 2.18, *p*<.032; and socialism vs. conservatism, *t*(113) = −4.50, *p*<.001, were all significant. Notably, the observed disambiguation was not symmetrical. The overall pattern was skewed to the right reflecting a general bias due to hemispheric asymmetry with verbal information presented to the right ear being processed more efficiently (e.g., [44]–[45]). As expected, for the remaining trials, words that were clearly presented to a particular auditory channel (100% and 50% volume) revealed no biases in channel disambiguation (conservatism words: right-channel, *M = *.92, *SD = *.12; left-channel *M = *.93, *SD = *.16, *t*(113) = .107, *p*<.915); socialism words: right-channel *M = *.92, *SD = *.13; left-channel *M = *.92, *SD = *.14, *t*(113) = −.313, *p*<.755); neutral words: right-channel *M = *.85, *SD = *.29; left-channel *M = *.89, *SD = *.27, *t*(113) = 1.302, *p*<.196). These findings are particularly important as they show that although the task of indicating on which channel the word was louder could prompt associations between the word meaning (socialism, conservatism) and the left/right auditory channels, participants showed almost perfect accuracy in indicating the channel where the word was presented louder, regardless its political meaning.

**Figure 2:**
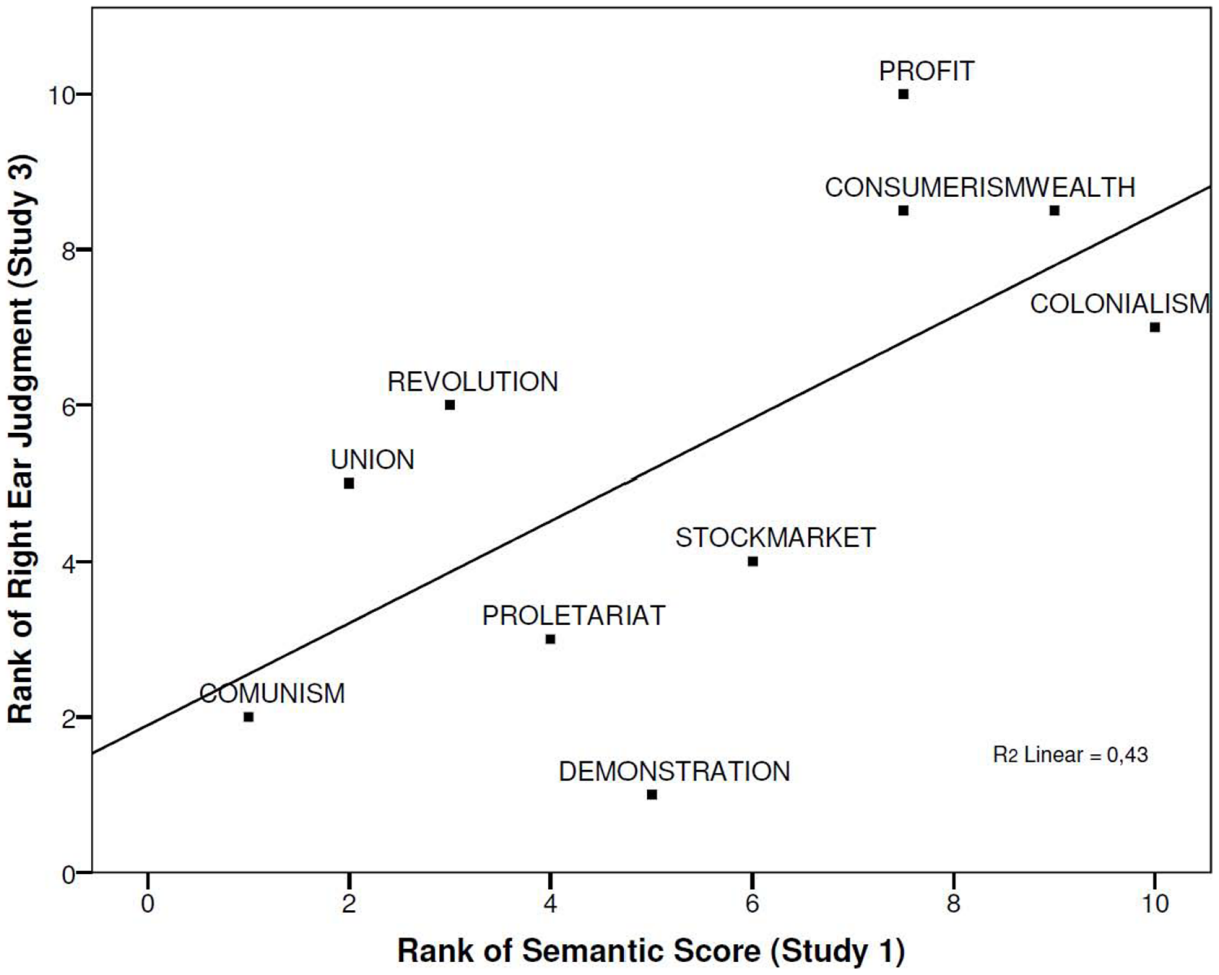
Ranked semantic judgments of the political stimuli in Study 1 plotted against their ranked percentage of right ear judgments in Study 3.

A linear regression revealed, as expected, that the systematic order of the semantic meaning of the stimuli predicted their right ear disambiguation, β = .655, *t*(9) = 2.45, *p*<.04 (R^2^ = .430). Thus, the more conservative the political meaning of the words, the more often these words were disambiguated to the right ear (Figure 2) indicating a remarkable convergence between the semantic and auditory rank ordering of the political terms. This mirrors the results of study 2 where a systematic overlap between semantic meaning and visual spatial position was observed.

**Figure 3:**
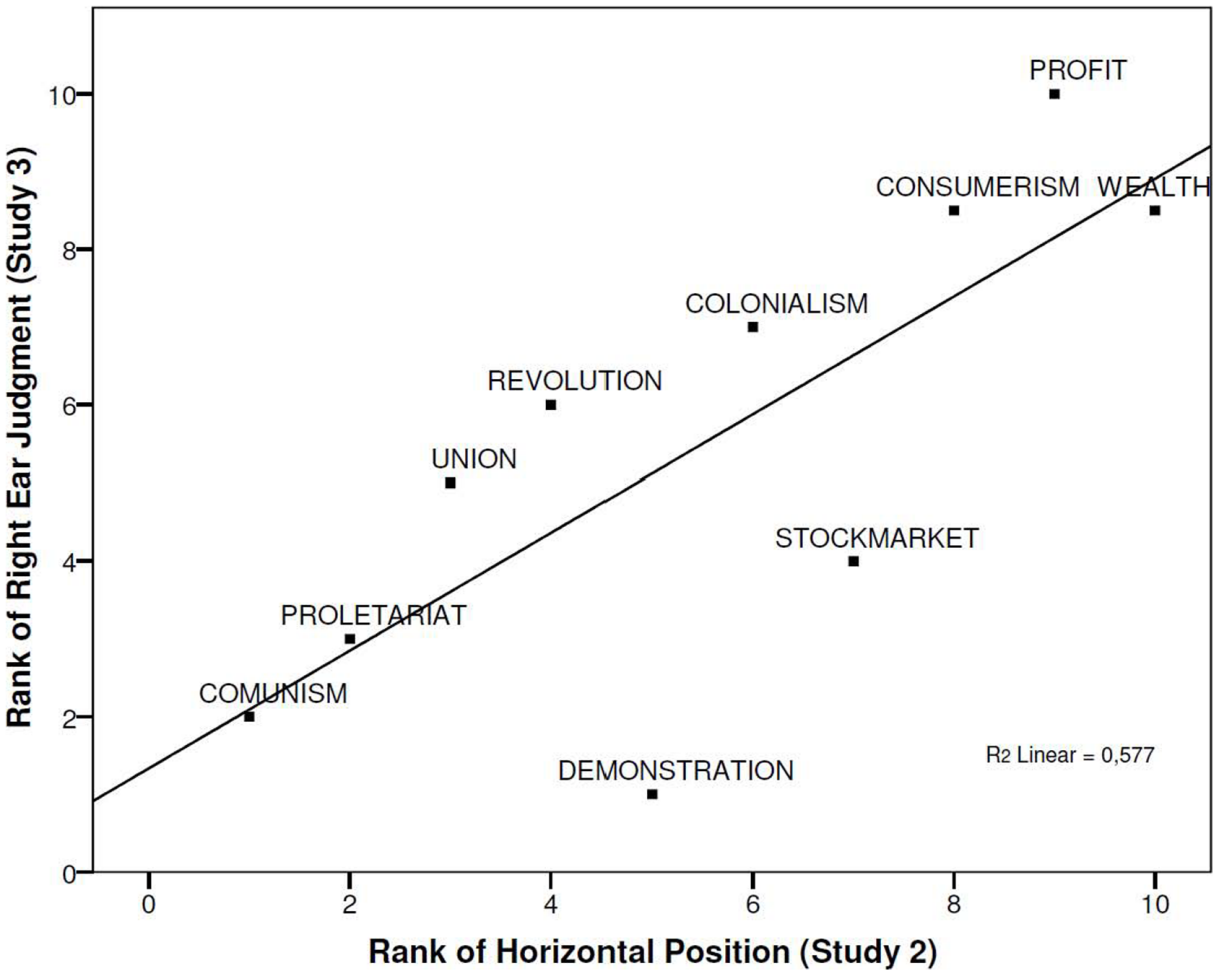
Ranked horizontal position of the political stimuli in Study 2 plotted against their ranked percentage of right ear judgments in Study 3.

To demonstrate the cross-modal convergence we conducted a further regression analysis between the rank ordering obtained in the visual task (Study 2) and the rank ordering resulting from the auditory disambiguation (Study 3). As expected, the spatial position of the stimuli (Study 2) predicted the auditory right ear judgments (Study 3), β = .760, *t*(9) = 3.31, *p*<.011 (R^2^ = .577). This pattern of results clearly indicates a substantial overlap between the spatial mapping of political words in the visual and auditory tasks (Figure 3). The more participants positioned politics-referent words to the right (Study 2), the more often these words were judged to be louder on the right channel (Study 3). This study constitutes the last element in the semantic, visual and auditory representation chain.

## General Discussion and Conclusions

Taken together these three studies reveal that the symbolic representation of an abstract category is also anchored in visual and auditory modalities. Furthermore, the three studies reveal a remarkable overlap between the three different representational orderings.

While it is possible to argue that the specific judgments in the semantic study and the visually driven placements in Study 2 are consciously produced, it is difficult to advance the same argument for Study 3. A process that escapes conscious access drives auditory disambiguation and it is unlikely that participants were aware of the systematicity they were producing. Nevertheless the overlap between the semantic, and the visual and the auditory tasks is remarkably high, sharing 70% and 43% of common variance respectively. This suggests that the multimodal representation of political concepts is highly homogeneously integrated.

Central to the research we have reported so far is the convergence between the three studies. We find that a spatial schema that is transmitted in a culture grounds political positions visually and auditorily. Moreover, the transduction is remarkable because it maintains the same spatial gradation across the semantic–symbolic representation and the visual and auditory modalities. In fact, we suggest that the distinction between symbolic and modality specific representations is most likely a mere analytic distinction that is experimentally induced rather than real. Obviously, the abstract category of political orientation is a multimodal representation whereby the distinction between semantic, visual and auditory constitutes different perspectives on the same representation. This is underlined by the remarkable average common variance (57%) between the three studies that have tapped on how political concepts are represented.

The broader ramifications of the current research are pertinent for the debate on how well embodiment accounts (e.g., simulation) deal with concrete (e.g., to kick, to pick, to lick) and abstract categories (e.g., morality, time, politics). Different authors adopt somewhat critical [35]–[36], [28]–[29], [37], but essentially convergent perspectives. At the one extreme are views (e.g., [36]) that regard language as a form of “dis-embodied” cognition, in particular with reference to abstract concepts. Dove, [35] argues that an embodiment approach has ‘limited reach’ when it comes to abstract concepts (e.g., [35], p. 428). The research we present here challenges this conclusion. An abstract category such as the politically charged socialist-conservative dimension is clearly multimodally grounded. In fact, the systematicity by which political concepts are represented visually and auditorily reflects the same regularity that is observed semantically. This suggests that there is a convergent and highly redundant regularity in the way in which abstract concepts are represented.

In concluding, we argue that the representation of concepts, concrete or abstract, is multimodal. Any single modality by which we capture the structure of a concept is likely to be reproduced in other modalities by which a concept can be represented, including what we regard as its symbolic representation. In fact, one provocative conclusion of the research we report here is that the claim that there is an opposition between symbolic representational and modality specific representations is misleading at best. Representations of concepts are multimodal and inseparably interwoven with their linguistic representations.
